# Breast Cancer Patients Have Greatly Benefited from the Progress in Molecular Oncology

**DOI:** 10.1371/journal.pbio.2000314

**Published:** 2016-09-29

**Authors:** Bernd L. Groner, Nancy E. Hynes

**Affiliations:** 1 Georg Speyer Haus, Institute for Tumor Biology and Experimental Therapy, Frankfurt am Main, Germany; 2 Friedrich Miescher Institute for Biomedical Research, Basel, Switzerland

## Abstract

Cancer research has become a global enterprise, and the number of researchers, as well as the cost for their activities, has skyrocketed. The budget for the National Cancer Institute of the United States National Institutes of Health alone was US$5.2 billion in 2015. Since most of the research is funded by public money, it is perfectly legitimate to ask if these large expenses are worth it. In this brief commentary, we recapitulate some of the breakthroughs that mark the history of breast cancer research over the past decades and emphasize the resulting benefits for afflicted women. In 1971, only 40% of women diagnosed with breast cancer would live another 10 years. Today, nearly 80% of women reach that significant milestone in most developed countries. This dramatic change has afforded breast cancer patients many productive years and a better quality of life. Progress resulted largely from advances in the understanding of the molecular details of the disease and their translation into innovative, rationally designed therapies. These developments are founded on the revolution in molecular and cellular biology, an entirely new array of methods and technologies, the enthusiasm, optimism, and diligence of scientists and clinicians, and the considerable funding efforts from public and private sources. We were lucky to be able to spend our productive years in a period of scientific upheaval in which methods and concepts were revolutionized and that allowed us to contribute, within the global scientific community, to the progress in basic science and clinical practice.

## Progress in Breast Cancer Research and Treatment

In 1971, only 40% of the women diagnosed with breast cancer could expect to survive this terrible finding for 10 years. This number has increased dramatically over the last 45 years. Today, nearly 80% of afflicted women reach this milestone in most developed countries (http://www.cancerresearchuk.org/health-professional/cancer-statistics/statistics-by-cancer-type/breast-cancer/survival). Such progress has allowed breast cancer patients to enjoy many more productive years and a better quality of life. What happened during that time interval, and what were the most important breakthroughs? The advances in understanding the basic molecular details of the disease were the driving force. These new insights were translated into innovative drug development and clinical studies and gradually led to progress in prevention, diagnosis, and treatment. However, 45 years ago, this path was far from clear for the experimental and clinical researchers who worked in the field. Dead ends, deviations, oversimplified concepts of the nature of the disease, and exaggerated expectations by the general public and the scientific community were constant companions of the research process. They were overcome by optimism, persistence, and diligence on the side of the researchers, driven by concurrent developments of methods and technologies, and finally enabled by considerable funding efforts of public and private agencies.

In the early 1970s, innovative concepts and technologies revolutionized all domains of biological investigations. Molecular cloning, DNA sequencing, and gene transfer procedures provided entirely new experimental approaches and transformed nearly all areas of biological research. Experimental and clinical cancer research were no exceptions. The discovery of tumorigenic viruses and the molecular dissection of these viruses led to the concept of “oncogenes.” The tumor viruses had hijacked “proto-oncogenes” from normal cells. Overexpression of such genes, or expression of slightly altered gain-of-function variants of the genes, was able to subvert intracellular signaling events in infected cells, cause their cellular transformation, and allow them to induce tumors [1]. Other crucial insights were provided by clinicians studying rare cancer predispositions. They found that certain cancer entities were inherited; only many years later did it become possible to identify and clone the mutated genes responsible [2]. Basic research in virology, molecular biology, and genetics converged and led to the disquieting conclusion that we all are carrying genes that have the potential, when mutated or overexpressed, to cause cancer. These breakthroughs, the identification of oncogenes, and insights into their mechanism of action fueled major hopes in the 1980s that human cancer could be understood and defeated within a foreseeable time frame.

The path of cancer research was never clearly marked. We were working in the 1980s on mouse mammary tumor virus (MMTV), which is transmitted through the milk of certain strains of mice, causing mammary cancer in their offspring. Many researchers, us included, expected that it would be possible to extrapolate the findings from mouse mammary tumors to human cancers. The hope was to find the human equivalent, i.e., a breast-cancer-causing virus, and use appropriate vaccination procedures or other antiviral strategies to protect women from viral infection and subsequently from the development of breast cancer. In the end, crucial differences between mice and men prevailed, and the human equivalent of MMTV is still elusive [3]. Was the work in vain? By no means; it provided new concepts to the field of steroid hormone action and resulted in the first definition of a steroid hormone response element present in a fragment of the MMTV genome [4]. This helped to explain why MMTV is strongly transcribed in the mammary gland, an organ in which multiple steroid hormones (corticoid steroids and sex steroids) are active and have essential roles in normal development and in cancer.

These insights, important for the understanding of the transcriptional regulation of gene expression by steroid hormones, found their way back to cancer research. Transcription factors, molecules that bind to specific DNA elements in promoter regions of particular target genes and induce the transcription of mRNA, were identified, among them the estrogen receptor. This receptor is normally present in the cell as a latent molecule, but can be activated through its association with a small hormone ligand, estrogen. A large fraction of breast cancer cells are utterly dependent on the transcriptional activating function of the estrogen receptor. This insight goes back to the observations made by the surgeon George Beatson more than 100 years ago. He showed that removal of the ovaries, the major source of estradiol, could lead to breast cancer regression.

Targeting the estrogen receptor became a therapeutic strategy in the 1960s. Variants of the estrogen molecule, which retain the ability to bind to the estrogen receptor but fail to activate it, were synthesized; one of these was named tamoxifen. These molecules mask the estrogen receptor and prevent estrogen action. Tamoxifen has become a widely used drug for the adjuvant treatment of patients in whom the tumor cells express the estrogen receptor [5]. Tamoxifen was administered to breast cancer patients after surgical removal of the primary tumor starting in the mid-1970s, and has had a tremendous impact on the number of women who survive for a long time after the first treatment of the disease. For many years, it has been the most valuable tool in the therapy of breast cancer and one of the first molecularly designed therapies [6].

The suppression of estrogen action in breast cancer cells has been complemented in recent years by inhibitors of aromatase. This enzyme participates in the local production of estrogen in tumor cells of postmenopausal patients. Counteracting the growth-promoting effects of local estrogen production is an effective weapon for clinicians and has been associated with relatively mild side effects for patients. Endocrine antagonists can be considered pioneers of the concept of “targeted therapy” [6]. Unfortunately, the therapeutic effects of antagonizing estrogen hormone action are often limited in duration. Cancer cells eventually progress and develop means to circumvent the inhibitory functions; they activate pathways that allow them to grow even in the presence of the inhibitors [7]. Serious efforts are underway to understand the mechanisms of resistance and to outsmart them in the next round of the “arms race.” Cytokine-regulated transcription factors, the so-called signal transducers and activators of transcription, STAT molecules, might become similarly promising targets during the next round of drug development [8].

Cells are constantly communicating with their environment, and many of the molecular signals and mechanisms constituting the communication networks between cells have been elucidated in the past decades. Cells are “wired;” they constantly recognize signals and send out signals. These events are encoded in molecular signal transduction cascades that eventually determine the cellular phenotypes. Crucial insights into the communication mechanisms of breast cancer cells, particularly through their cell surface receptor signaling, have contributed greatly to progress in breast cancer therapy. Differently from the estrogen receptor, which acts as a transcription factor in the nucleus, cell surface receptors establish links between the extracellular environment and the intracellular signal transduction events. These receptors are anchored in the cell membrane and contain extracellular and intracellular domains. They sense signal molecules, e.g., growth factors or cytokines, through their extracellular domain and generate intracellular signals that, through a cascade of biochemical reactions, regulate cell survival and cell growth. The hyperactivity of such receptors can be a cause of cancer. In the 1980s and 1990s, researchers identified a large number of such receptors and characterized the intracellular signals emanating from their activation.

One particular receptor, ErbB2, a member of the receptor tyrosine kinase family, was found in the mid-1980s to be overexpressed in about 25% of breast cancer cases. Importantly, clinical results supported the relevance of overexpressed ErbB2, which has become an extremely important drug target that has proved most beneficial for cancer therapy [9]. Since the receptor is accessible from the extracellular side, antibodies were derived that bind to its extracellular domain and antagonize its function. Such antibodies were administered to breast cancer patients in whom ErbB2 overexpression had been identified, and this treatment was found to prolong the overall survival of metastatic breast cancer patients. ErbB2-targeting antibodies soon became a complementary therapeutic tool to nuclear hormone receptor antagonists and chemotherapeutic agents.

The “story” of ErbB2 targeting also became one of the first examples for the strategy in which molecular diagnosis and targeted therapy have been successfully combined. ErbB2 expression can be determined in breast tumor tissues biopsies, and only the subpopulation of patients in whom the receptor is strongly expressed have a good chance to benefit from the antibody treatment. The prospective identification of patients most likely to respond to a particular targeted drug has important implications, not only for the choice of first-line therapy, but also for health economics. Subsequently, therapeutic strategies based on different molecular principles have been used in combination [10]. For example, antibodies against cell surface receptors and cytotoxic chemotherapeutic agents were found to cooperate and to further improve treatment outcome [11]. This was a most welcome observation but was not necessarily expected.

Insights into the intracellular signaling events triggered by cell surface receptor activation opened another avenue for treatment advances. Most growth factor receptors set in motion an intracellular signaling cascade through the activation of their tyrosine kinase in the intracellular domain; tyrosine kinases are enzymes that phosphorylate substrate molecules on tyrosine residues. Agents interfering with the enzymatic activity of specific tyrosine kinases can block the signaling pathways activated by the receptors, for example, ErbB2. In addition, since many of the downstream steps in the signaling pathways involve protein kinases, researchers could develop targeted drugs for nearly every step in the signal transduction cascades. Such drugs, in combination with advanced molecular diagnostics that provide insights into the deregulation of signaling in particular cancer cells, will further improve our therapeutic capabilities [12].

Despite these achievements, we have certainly not reached the final goal, which is the prevention and cure of cancer, and there are many open questions. Is further progress to be expected? From which area of research will it come? A number of research directions appear most promising and are giving cause for optimism. Large-scale sequencing efforts provide high-resolution views into the mutational events in breast cancer cells. Numerous base substitutions, insertions, deletions, and structural variations have been found. From these genetic aberrations, we can deduce the deregulation of crucial biochemical processes in cancer cells. Recently, the entire genomes of 560 breast cancer cases have been deciphered and 93 altered “driver” genes have been identified. These genes are most likely causally involved in the genesis of the disease. Their identification also deepens our understanding of the causes of breast cancer and provides new targets for drugs that interfere with cancer growth [13]. These promising developments are being complemented by further progress in basic biological research. The exploitation of the potential of immune cells to eliminate tumor cells has been a hope for decades, and it is now finally nearing fruition. Insights into the intricate regulation of the activities of cytotoxic T cells and NK cells allow us to manipulate these processes and to specifically direct the immune cells against tumor cells. This strongly enhances our therapeutic capabilities [14]. In the near future, we also hope that insights into the gene regulatory effects of miRNAs, the manipulation of epigenetic processes in tumor cells, and preventive vaccination will serve to further improve our therapeutic tools.

However, despite these therapeutic advances, a most disturbing aspect of tumor biology remains. The decline in breast cancer mortality over the last 45 years is impressive, but the incidence still continues to rise. For this reason, progress in cancer prevention becomes a most urgent task. Geneticists have uncovered a few of the determinants that predispose women to breast cancer. Functional variants of the *BRCA1* and *BRCA2* genes account for 2%–3% of all cases of breast cancer. The early identification of women who are affected with a *BRCA* mutation can guide the management of cancer risk and can have a significant impact on the morbidity and mortality of *BRCA* carriers. The current risk management recommendations for breast cancer comprise preventive therapy with selective estrogen receptor modulators (SERMs), and considerable reductions in overall breast cancer incidence have been observed. Despite the decreased probability for developing breast cancer, only about 15% of the women made use of the preventive therapy even after genetic counseling [15].

Will we succeed in taking the final step and increase the 10-year survival rate to 100%? The prospects are encouraging. The development of targeted agents, immunological strategies, and preventive measures will individually, and in combination, lead to improved therapies and to a further decline in incidence rates. The advances we've yet to see, like the advances in treatment and the gains in life expectancy and quality of life that breast cancer patients experience today, will come from the efforts and insights provided by dedicated scientists and clinicians. They could not have been planned 45 years ago and reflect much serendipity and ingenuity, defining the way we do basic research. We may not know exactly what discoveries will ultimately help patients, and society at large, but history shows that money spent on basic cancer research is money well spent.

## References

[1] Bishop JM. Viral oncogenes. Cell. 1985; 42: 23–38. 2990725 10.1016/s0092-8674(85)80098-2

[2] Malkin D, Garber JE, Strong LC, Friend SH. CANCER. The cancer predisposition revolution. Science. 2016; 352: 1052–1053. 27230363 10.1126/science.aag0832

[3] Salmons B, Lawson JS, Gunzburg WH. Recent developments linking retroviruses to human breast cancer: infectious agent, enemy within or both? J Gen Virol. 2014;95: 2589–2593. 10.1099/vir.0.070631-0 25217613

[4] Hynes NE, Kennedy N, Rahmsdorf U, Groner B. Hormone-responsive expression of an endogenous proviral gene of mouse mammary tumor virus after molecular cloning and gene transfer into cultured cells. Proc Natl Acad Sci U S A. 1981; 78: 2038–2042. 6264458 10.1073/pnas.78.4.2038 PMC319278

[5] Jordan VC. Tamoxifen: catalyst for the change to targeted therapy. Eur J Cancer. 2008; 44: 30–38. 18068350 10.1016/j.ejca.2007.11.002 PMC2566958

[6] McNamara KM, Guestini F, Sakurai M, Kikuchi K, Sasano H. How far have we come in terms of estrogens in breast cancer? Endocr J. 2016; 63: 413–424. 10.1507/endocrj.EJ16-0022 27020038

[7] De Marchi T, Foekens JA, Umar A, Martens JW. (2016) Endocrine therapy resistance in estrogen receptor (ER)-positive breast cancer. Drug Discov Today. 2016;7: 1181–1188. 10.1016/j.drudis.2016.05.012 27233379

[8] Groner B, Hennighausen L. The versatile regulation of cellular events by Jak-Stat signaling: from transcriptional control to microtubule dynamics and energy metabolism. Horm Mol Biol Clin Investig. 2012;10: 193–200. 10.1515/hmbci-2012-0010 25436675

[9] Hynes NE. ErbB2: From an EGFR Relative to a Central Target for Cancer Therapy. Cancer Res. 2016; 76: 3659–3662. 10.1158/0008-5472.CAN-16-1356 27371737

[10] Tolaney SM, Barry WT, Dang CT, Yardley DA, Moy B, et al Adjuvant paclitaxel and trastuzumab for node-negative, HER2-positive breast cancer. N Engl J Med. 2015; 372: 134–141. 10.1056/NEJMoa1406281 25564897 PMC4313867

[11] Hurvitz SA, Andre F, Jiang Z, Shao Z, Mano MS, et al Combination of everolimus with trastuzumab plus paclitaxel as first-line treatment for patients with HER2-positive advanced breast cancer (BOLERO-1): a phase 3, randomised, double-blind, multicentre trial. Lancet Oncol. 2015; 16: 816–829. 10.1016/S1470-2045(15)00051-0 26092818

[12] Lee JJ, Loh K, Yap YS. PI3K/Akt/mTOR inhibitors in breast cancer. Cancer Biol Med. 2015; 12: 342–354. 10.7497/j.issn.2095-3941.2015.0089 26779371 PMC4706528

[13] Nik-Zainal S, Davies H, Staaf J, Ramakrishna M, Glodzik D, et al Landscape of somatic mutations in 560 breast cancer whole-genome sequences. Nature. 2016; 534: 47–54. 10.1038/nature17676 27135926 PMC4910866

[14] Carvalho S, Levi-Schaffer F, Sela M, Yarden Y. Immunotherapy of cancer: from monoclonal to oligoclonal cocktails of anti-cancer antibodies: IUPHAR Review. Br J Pharmacol. 2016;173: 1407–1424. 10.1111/bph.13450 26833433 PMC4831314

[15] Smith SG, Sestak I, Forster A, Partridge A, Side L, et al Factors affecting uptake and adherence to breast cancer chemoprevention: a systematic review and meta-analysis. Ann Oncol. 2016; 27: 575–590. 10.1093/annonc/mdv590 26646754 PMC4803450

